# Nitrogen Fertilization Coupled with Zinc Foliar Applications Modulate the Production, Quality, and Stress Response of *Sideritis cypria* Plants Grown Hydroponically Under Excess Copper Concentrations

**DOI:** 10.3390/plants14050691

**Published:** 2025-02-24

**Authors:** Nikolaos Tzortzakis, Giannis Neofytou, Antonios Chrysargyris

**Affiliations:** Department of Agricultural Sciences, Biotechnology and Food Science, Cyprus University of Technology, 3603 Limassol, Cyprusa.chrysargyris@cut.ac.cy (A.C.)

**Keywords:** copper stress, soilless culture, micronutrients, antioxidant capacity, mineral uptake

## Abstract

The demand for medicinal and aromatic plants (MAPs) has grown significantly in recent years, due to their therapeutic value. Among these, *Sideritis cypria* Post is a promising yet under-evaluated species. Existing research assessing the effects of nitrogen (N) fertilization, zinc (Zn) foliar applications, and toxic copper (Cu) concentrations often overlooks MAPs such as *S. cypria*. Additionally, the interactions among these parameters, as well as their combined roles in MAPs plant physiology and secondary metabolite biosynthesis, have yet to be fully elucidated. In this study, hydroponically grown *S. cypria* plants were cultivated using nutrient solutions (NSs) with different N (75, 150, and 300 mg L^−1^) and Cu (5 and 100 μM) levels, combined with foliar spraying (0 and 1.74 mM Zn), to evaluate the growth, mineral uptake, secondary metabolites production and stress response. N levels at 75 and 150 mg L^−1^ resulted in increased dry matter content, whereas fresh biomass production was preserved. Foliar Zn applications enhanced chlorophylls and antioxidants, contingent upon N and Cu in the NS. Increased N accumulation was observed via the increase in N in the NS, while foliar Zn enhanced its uptake at moderate N levels. Excess Cu stimulated its accumulation, while a reduction was observed with foliar Zn at low and high N levels. Excess Cu increased lipid peroxidation (MDA) at low and moderate N in the NS, while foliar Zn decreased both MDA and hydrogen peroxide, contingent upon Cu and N levels. Low-to-moderate N in the NS can be applied under excess Cu without compromising the yield, quality, and safety of *S. cypria* plants, while foliar Zn can modulate the stress response of plants under excess Cu and the production of secondary metabolites. These results may be utilized for optimizing nutrient management strategies for the cultivation of MAPs, contributing to conservation efforts by supporting the cultivation of endemic species like *S. cypria*, considering the potential benefits of Zn foliar applications under Cu-contaminated conditions.

## 1. Introduction

Consumer demand for medicinal and aromatic plants (MAPs) has increased over the years due to the awareness of their potential benefits. To meet the growing demand, the agro-industrial and pharmaceutical sector require consistent yield and quality [1,2]. However, various factors, such as climatic conditions, seasonal changes, pathogen levels, pests, and the status of soils, affect the amount of secondary metabolites produced by MAPs, complicating the standardization of their cultivation [3]. To address this, protected cultivation and soilless culture systems (SCSs) offer effective strategies for ensuring stable MAP production [4]. In SCS, a crucial parameter is the optimization of the nutrient solution composition, which can have a substantial impact on MAPs’ growth. Furthermore, tailor-made fertilization strategies can be critical for the synthesis of components with medicinal attributes [5].

The genus *Sideritis*, belonging to the Lamiaceae family, encompasses 150 species commonly known as “ironwort” or “mountain tea” [6,7]. These species are primarily found in the Mediterranean region, and they are recognized for their medicinal properties due to the presence of chemical components such terpenes, flavonoids, and coumarins [8]. Among these species, *Sideritis cypria* Post, a perennial herb endemic to Cyprus, is traditionally used against stomach disorders, headaches, and the common cold, as infusions; however, it largely remains underexplored both commercially and scientifically [9,10]. The lack of comprehensive scientific research presents an obstacle to unlocking the full potential of various MAPs [4], including *S. cypria*, as a limited number of studies have been conducted to investigate its cultivation [10,11].

Nitrogen (N) fertilization is often associated with increased yields and is widely employed in the cultivation of various MAPs [12]. In addition, N is critical in the chemical and medicinal composition of MAPs [13]. As a fundamental constituent of proteins, chlorophyll, amino acids, and alkaloids, N directly influences key bioactive compounds [14]. Amino acids, in particular, serve in the biosynthesis of numerous essential oil (EO) constituents, which contribute to the medicinal properties of MAPs [15]. However, excess N application often leads to N losses (e.g., nitrate leaching), increased nitrous oxide emissions, and soil degradation [16,17]. In addition, increased and unbalanced N fertilization may negatively affect the plant’s metabolism and medicinal status, as evidenced in *Ocimum basilicum* L. [18] and *Salvia miltiorrhiza* Bunge [19]. Nonetheless, N deprivation exerts a detrimental effect on plant physiology, restricting photosynthesis, transpiration, and stomatal conductance, while N-starved plants may also exhibit increased synthesis of secondary metabolites [3].

Soil pollution by heavy metals has become a significant environmental concern, particularly due to the heavy industrialization and agrochemicals use [20]. This has led to the accumulation of heavy metals such as copper (Cu). Copper is an essential plant micronutrient, constituting a redox-active transition element and influencing photosynthesis, C and N metabolism, respiration, and oxidative stress protection [21]. Although essential, in excess, Cu can become hazardous to plants, leading to phytotoxicity [22,23]. Elevated Cu can disrupt plant growth by interfering with metabolic processes due to the increased production of reactive oxygen species (ROS), influencing membrane function and permeability [24]. Damage occurs due to redox cycles between Cu^2+^ and Cu^+^, inducing the production of hydroxyl radicals. Additionally, oxidative damage occurs as copper binds to proteins, disrupting cellular processes [25]. Interestingly, Cu is also involved in the nitrogen metabolism, where optimal concentrations enhance the plants’ utilization of N in soils [26]. However, increased levels of Cu may also undermine the uptake of ammonium (NH_4_^+^) and nitrate (NO_3_^−^) through the inhibition of N assimilation-related enzymatic activities [27].

Zinc (Zn) is another essential micronutrient that has roles in the structure and regulation of various enzymes and proteins via its influence in the carbohydrate metabolism, photosynthesis, protein metabolism, auxin metabolism, and the integrity of biological membranes [28,29]. Zinc can alleviate the harmful effects of environmental stressors, as it increases active oxygen scavenging substances and reduces lipid peroxidation [30,31]. Interestingly, Zn also exhibits antagonistic interactions with Cu, which significantly influences their uptake, bioavailability, and quantitative status [29]. For this reason, the interaction between Zn and Cu may reduce the effects of Cu toxicity [25]. Moreover, the co-supply of Zn and N can prompt increased root-to-shoot translocation and distribution of both nutrients, significantly influencing plant growth [32]. In addition, agronomic Zn biofortification has become more prevalent, as a means of overcoming human health concerns related to Zn deficiency [33].

While N is essential for biomass production and enzymatic functions, in the presence of excessive Cu, its uptake is disrupted, and oxidative stress is induced. Zinc may alleviate this response, and although excess N levels may enhance its mobility, they could also exacerbate Cu toxicity under certain conditions. Despite their importance, the interactions between these micronutrients and their collective effect on plant growth, in conjunction with variable N fertilization, remain relatively overlooked, particularly in under-researched species such as *S. cypria*. The objective of this study was to investigate the impact of various N levels in the nutrient solution on the growth and physiology of *S. cypria* plants grown in soilless cultivation systems, while simultaneously investigating the impact of elevated Cu concentrations and foliar Zn applications. As this examination explores these effects, it is hypothesized that excess Cu in the hydroponic NSs may negatively affect the growth and physiological performance of *S. cypria*, while combined foliar Zn applications are expected to mitigate Cu-induced stress. In addition, varying N levels are expected to modulate the effects of both Cu and Zn, potentially influencing the overall plant growth, nutrient status, and secondary metabolite production.

## 2. Results

Table 1 presents an overview of the effect of the different N levels (N), Zn foliar applications (Zn), and Cu applications (Cu); their first-order interactions (N × Cu, N × Zn, Cu × Zn); and their second-order interaction (N × Cu × Zn) on *S. cypria* plant growth and physiology. N levels affected Chl a, Total Chl, Total Car, leaf Zn, and root Cu at *p* < 0.05. Also, N levels affected SPAD; DM; Fv Fm^−1^; leaf N, K, Na, and Fe; root N, P, K, Na, and Zn; phenols; flavonoids; and H_2_O_2_ at *p* < 0.001. Cu impacted flavonoids at *p* < 0.01. And it impacted DM; Fv Fm^−1^; leaf Na and Cu; and root N, K, Na, and Cu at *p* < 0.001. Zn impacted root Fe at *p* < 0.05; and DM, leaf N, and root Zn at *p* < 0.01. It also impacted leaf P and Na; root P, K, and Na; phenols; DPPH; FRAP; ABTS; flavonoids; and MDA at *p* < 0.001. The N × Cu interaction affected Total Chl, leaf Fe, FRAP, and H_2_O_2_ at *p* < 0.05; Chl a and root K, Na, Cu, and Zn at *p* < 0.01; and Total Car, leaf N and Na, root N and P, phenols, DPPH, ABTS, flavonoids, and MDA at *p* < 0.001. The N × Zn interaction affected leaf Cu and Zn, and root Cu and Fe at *p* < 0.05; Fv Fm^−1^, Chl b, and leaf N at *p* < 0.01; and Chl a, Total Chl, Total Car, leaf P and Na, root macronutrients and Zn, phenols, DPPH, FRAP, ABTS, flavonoids, and H_2_O_2_ at *p* < 0.001. The Cu × Zn interaction affected Chl a, Total Chl, Total Car, leaf Cu, FRAP, and MDA at *p* < 0.05; and root N and K, phenols, DPPH, and ABTS at *p* < 0.001. The N × Cu × Zn interaction affected Chl b, root P, and Cu at *p* < 0.05; Total Chl, root Zn, and ABTS at *p* < 0.01; and Chl a, Total Car, leaf Cu, root K and Na, phenols, DPPH, FRAP, flavonoids, and H_2_O_2_ at *p* < 0.001.

**Figure 1:**
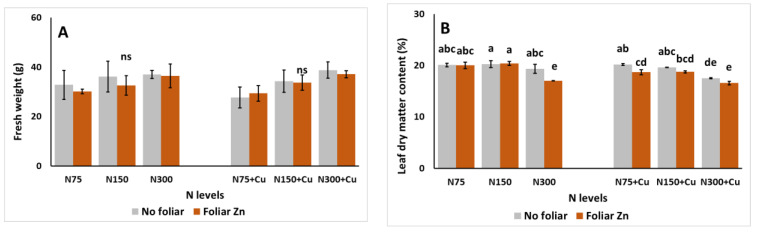
The effect of N levels (N75, 75 mg L^−1^; N150, 150 mg L^−1^; and N300, 300 mg L^−1^), Zn foliar application (0 and 1.74 mM Zn), and Cu application (no additional, 5 μM Cu; and with additional Cu, 100 μM Cu) on the fresh weight (FW; g) (**A**) and dry matter (DM; %) (**B**) of *Sideritis cypria* plants grown in soilless cultivation. Significant differences (*p* < 0.05) among the different applications are indicated by different letters. ns: not significant.

The effects of the examined parameters on *S. cypria* upper biomass production (fresh weight, FW) and dry matter content (DM) are presented in Figure 1. Plant FW remained unaffected by the examined parameters. However, DM was significantly influenced by the examined parameters. The lowest values were attained under the application of N300 and foliar Zn (1.74 mM). However, these values did not differ significantly with no foliar Zn (0 mM) and Cu at 100 μM. Plants grown using the N75 NS and 100 μM Cu exhibited decreased DM with the application of foliar Zn, compared to foliar spraying with pure water. Finally, plants grown using the N150 exhibited decreased DM with the combined application of Cu at 100 μM and foliar Zn, compared to plants treated with Cu at 5 μM.

**Figure 2:**
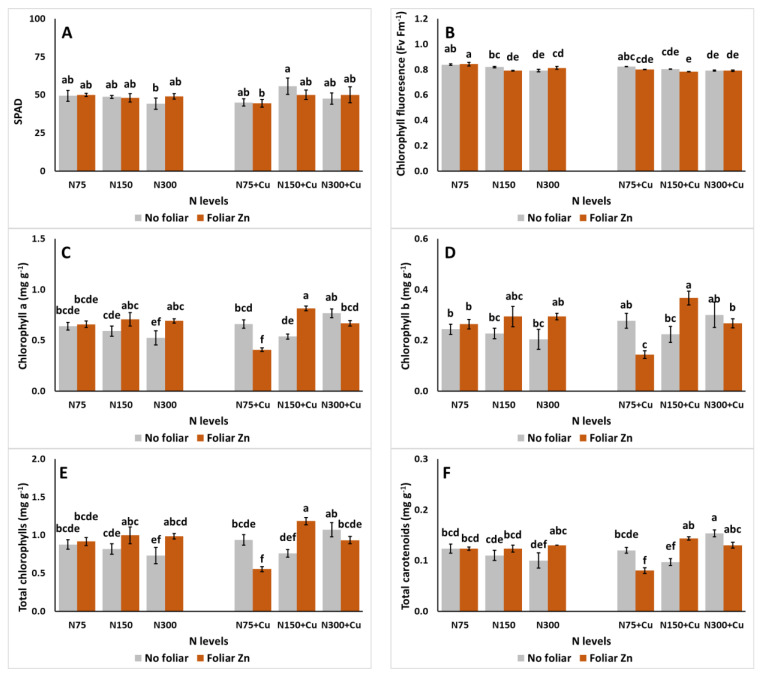
The effect of N levels (N75, 75 mg L^−1^; N150, 150 mg L^−1^; and N300, 300 mg L^−1^), Zn foliar application (0 and 1.74 mM Zn), and Cu application (no additional, 5 μM Cu; and with additional Cu, 100 μM Cu) on leaf SPAD (**A**), chlorophyll fluorescence (Fv Fm^−1^) (**B**), chlorophyll a (Chl a; mg g^−1^) (**C**), chlorophyll b (Chl b; mg g^−1^) (**D**), total chlorophylls (Total Chl; mg g^−1^) (**E**), and total carotenoids (Total Car; mg g^−1^) (**F**) of *Sideritis cypria* plants grown in soilless cultivation. Significant differences (*p* < 0.05) among the different applications are indicated by different letters.

Leaf phytochemistry features, as affected by the examined parameters, are presented in Figure 2. SPAD (Figure 2A) was decreased with the applications of N75 with 100 μM Cu and foliar spraying with Zn (1.74 mM), and N300 with 5 μM Cu and foliar spraying without Zn (0 mM). For chlorophyll fluorescence (Fv Fm^−1^) (Figure 2B), the highest value was recorded for plants that were subjected to the co-application of N75, 5 μM Cu, and foliar spraying with Zn, which was significantly higher than the rest of the treatments, excluding intermediate values, such as those attained with the co-application of N75, foliar spraying without Zn, and with 5 or 100 μM Cu. In contrast, the lowest Fv Fm^−1^ was presented with the co-application of N150, 100 μM Cu, and foliar spraying with Zn. For Chl a (Figure 2C), the highest value was recorded with the co-application of N150, 100 μM Cu, and foliar spraying with Zn, which was significantly greater than the lowest value attained with the co-application of N75, 100 μM Cu, and foliar spraying with Zn. A similar finding is observed in both Chl b (Figure 2D) and Total Chl (Figure 2E), while Total Car (Figure 2F) was mostly negatively affected with lower N levels in the NS.

**Figure 3:**
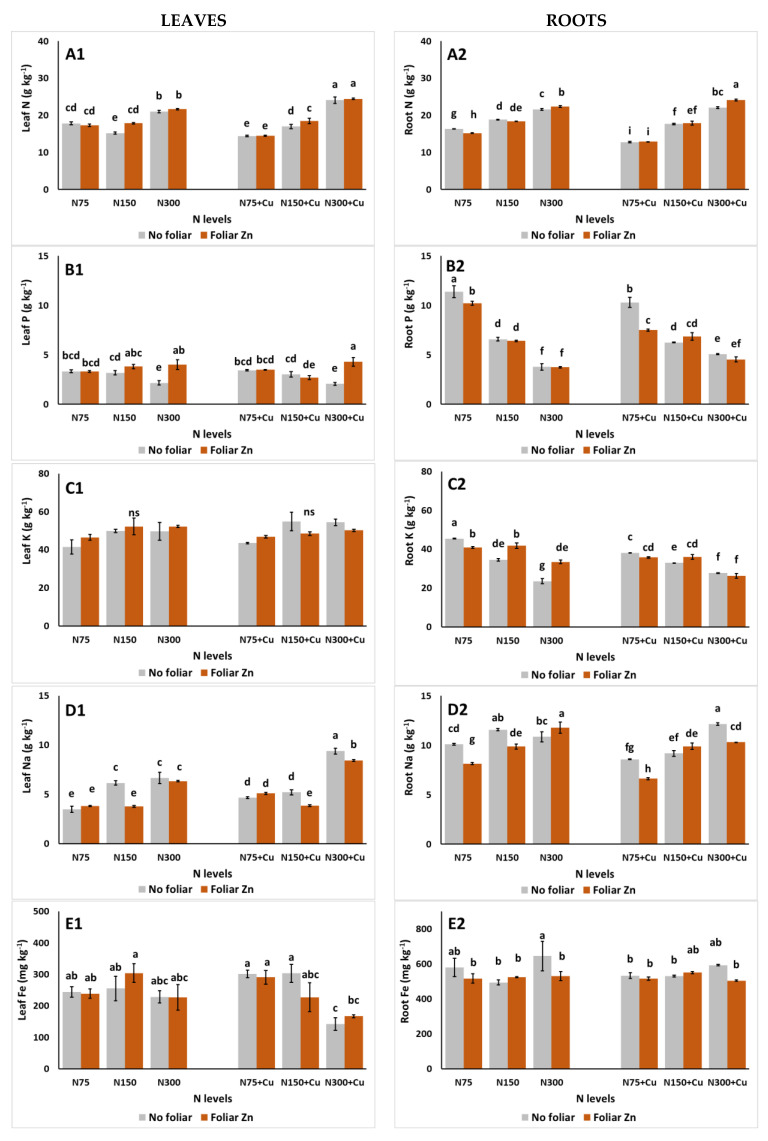

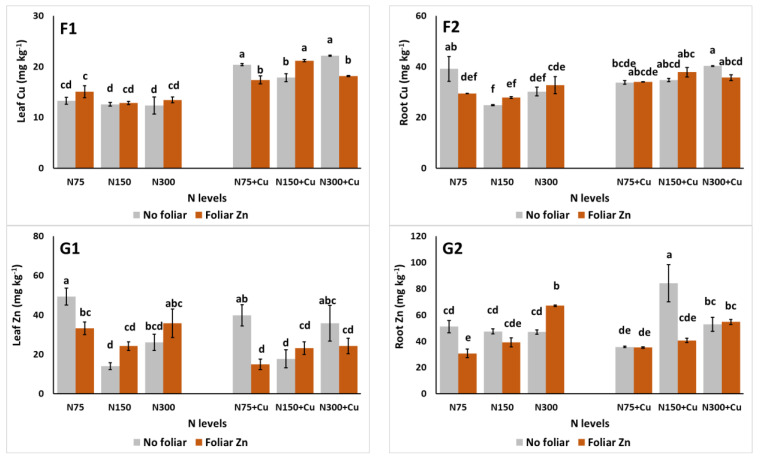
The effect of N levels (N75, 75 mg L^−1^; N150, 150 mg L^−1^; and N300, 300 mg L^−1^), Zn foliar application (no foliar, 0 mM Zn; and foliar Zn, 1.74 mM Zn), and Cu application (no additional, 5 μM Cu; and with additional Cu, 100 μM Cu) on leaf and root N (**A1**,**A2**), P (**B1**,**B2**), K (**C1**,**C2**), Na (**D1**,**D2**), Fe (**E1**,**E2**), Cu (**F1**,**F2**), and Zn (**G1**,**G2**), respectively, of *Sideritis cypria* plants grown in soilless cultivation. Significant differences (*p* < 0.05) among the different applications are indicated by different letters. ns: not significant.

The impacts of the examined parameters on the leaf and root mineral content are examined in Figure 3. In leaves, the N content was increased at the highest N concentration in the NS (N300), while this increase was amplified with the co-application of N300 and Cu at 100 μΜ (Figure 3A1). Similarly, in roots, N was accumulated with the application of N300, while the highest N content was observed with the co-application of N300, 100 μM Cu, and the foliar application of Zn (Figure 3A2). In both leaves and roots, the lowest accumulation of N was observed with the co-application of N75 and 100 μM Cu. P content in leaves was the lowest with the application of N300 without foliar Zn, while the application of Zn in foliar spraying led to a marginal increase in P with the use of the same NS (Figure 3B1). In the case of root P content, it was generally decreased by the increase in N in the NS (Figure 3B2), while no significant influences were observed for Cu and Cu × Zn applications (Table 1). Leaf K remained unaffected by the examined treatments (Figure 3C1), while root K was the lowest and highest with the application of N300 and N75, respectively, under 5 μM Cu and without foliar Zn (Figure 3C2). In the case of Na in leaves, it was generally increased with the highest N in the NS (N300), while the highest values were observed with the application N300, 100 μΜ Cu, and no foliar Zn. Interestingly, for the N150 treatments, Na was decreased with the foliar application of Zn (Figure 3D1). A similar trend was observed in the accumulation of Na in roots, as it decreased with the foliar application of Zn in all treatments, excluding the case of N150 and 100 μM Cu, as no significant differences were observed (Figure 3D2).

In terms of micronutrients, the Fe content of leaves was significantly decreased with the application of 100 μM Cu, under the N300 NS, compared to the application of lower Cu levels (5 μM) (Figure 3E1). In addition, foliar Zn caused a decrease in root Fe in the N300 NS with 5 μM Cu (Figure 3E2). The Cu content of leaves was significantly increased by the application of 100 μM Cu, compared to the application of normal Cu levels (5 μM) in the NS (Figure 3F1). Foliar Zn applications led to the decrease in Zn content of leaves using the N75 NS, while a significant increase was evident using the N150 NS with 5 μM Cu. A decrease was also observed when excess Cu was applied, using the N75 NS with foliar Zn (Figure 3G1). The highest root Zn was recorded for plants treated with N150, 100 μM Cu, and no foliar Zn. Finally, root Zn was decreased with the application of foliar Zn, using the N75 NS and 5 μM Cu, while the opposite occurred using the N300 NS and 5 μM Cu (Figure 3G2).

**Figure 4:**
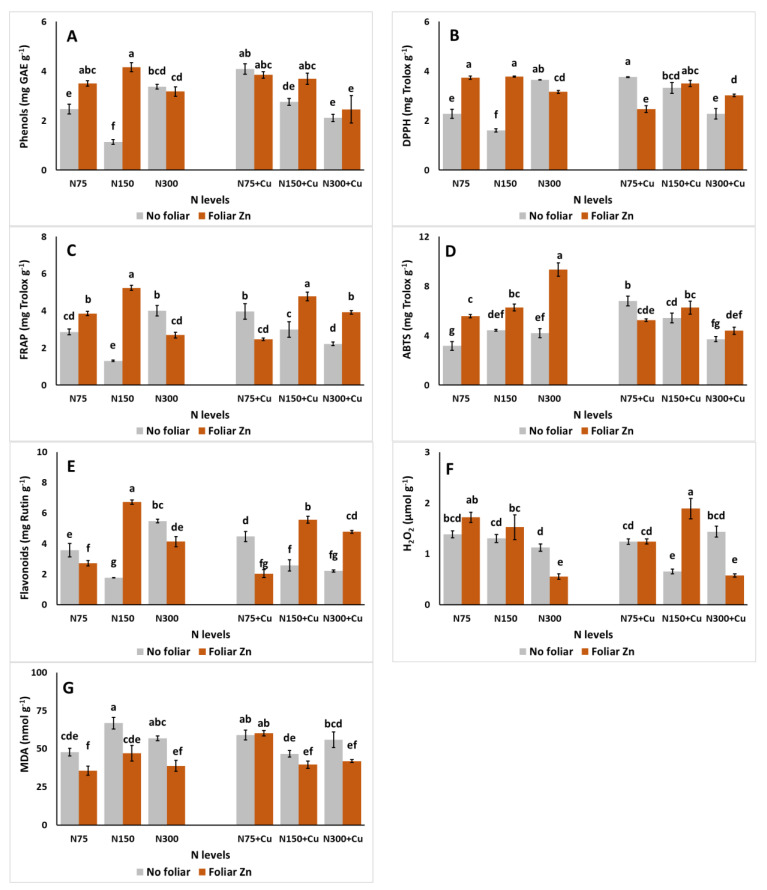
The effect of N levels (N75, 75 mg L^−1^; N150, 150 mg L^−1^; and N300, 300 mg L^−1^), Zn foliar application (no foliar, 0 mM Zn; and foliar Zn, 1.74 mM Zn), and Cu application (no additional, 5 μM Cu; and with additional Cu, 100 μM Cu) on phenols (**A**), DPPH (**B**), FRAP (**C**), ABTS (**D**), flavonoids (**E**), H_2_O_2_ (**F**), and MDA (**G**) of *Sideritis cypria* plants grown in soilless cultivation. Significant differences (*p* < 0.05) among the different applications are indicated by different letters.

The effects of the N-modified nutrient solutions, different Cu levels, and foliar Zn on total phenols content, antioxidant capacity, and flavonoids of *S. cypria* plants are presented in Figure 4. The lowest phenolics (Figure 4A) were recorded in plants cultivated with N150, 5 μM Cu, and no foliar Zn. The application of foliar Zn, however, in the same treatment, resulted in a significant increase in phenolics. The same was observed with the applications of N75 with 5 μM Cu, and N150 with 100 μM Cu. In the case of N75 and N150, the application of increased Cu led to an increase in total phenolics when no foliar Zn was applied. Similar observations can be viewed in terms of antioxidant capacity, assayed by DPPH (Figure 4B), FRAP (Figure 4C), and ABTS (Figure 4D). Interestingly, DPPH and FRAP were increased with the application of foliar Zn in the treatment with N300 and 100 μM Cu, whereas the inverse occurred with the lower Cu dose (5 μM). For ABTS, the highest values were observed with the application of N300 with 5 μM Cu and foliar Zn, compared to the rest of the treatments, whereas the lowest was observed with the application of N75 with 5 μM Cu and no foliar Zn. Finally, the highest flavonoids (Figure 4E) were observed with the application of N150 with 5 μM Cu and foliar Zn compared to the rest of the treatments, whereas the lowest were observed with the equivalent treatment with no foliar Zn application.

The effects on hydrogen peroxide (H_2_O_2_) and lipid peroxidation in terms of MDA are presented in Figure 4. In the case of H_2_O_2_ (Figure 4F), a significant increase was observed with the application of N150, 100 μM Cu, and foliar Zn, compared to the rest of the treatments, excluding N75, 5 μM Cu, and foliar Zn. In addition, the lowest H_2_O_2_ levels were observed in plants treated with the co-application of N300 with 5 or 100 μM Cu and foliar Zn, and N150 with 100 μM Cu and no foliar Zn. In the case of MDA (Figure 4G), decreased values were observed with the application of foliar Zn; in N75, N150, and N300 combined with 5 μM Cu; and N300 with 100 μM Cu.

Linear correlation coefficients were calculated to demonstrate the contribution of the N (Supplementary Table S2), Cu (Supplementary Table S3), and Zn (Supplementary Table S4) concentrations for each individual factor. Regarding N levels in the NS, there was a positive correlation with N (75–150–300 mg L^−1^) concentrations and leaf minerals (N, Na, and Zn), and root N; and a negative correlation with leaf Fe, root P, root K, dry matter content, and H_2_O_2_ content (Supplementary Table S2). Regarding Cu levels in the NS, there was a positive correlation with Cu (5 and 100 μM) concentrations and leaf Cu and root Cu, and a negative correlation with chlorophyll fluorescence and dry matter content (Supplementary Table S3). Regarding Zn foliar applications, there was a positive correlation with Zn (0 and 1.74 mM) concentrations and leaf P, phenols, and antioxidant (FRAP and ABTS) content, and a negative correlation with MDA (Supplementary Table S4).

## 3. Discussion

Soilless cultivation provides control over nutrient management, allowing for the optimization of plant growth. This is particularly important for specialty crops, including MAPs, where consistent yield and quality are of utmost importance [4]. Nitrogen fertilization remains an important parameter for the growth of MAPs, as it can directly affect the yield and quality of the final product [12]. However, in the case of *S. cypria*, knowledge regarding this topic is scarce [10]. Moreover, nutrient interactions represent an intricate aspect of plant nutrition, as the dynamics of a nutrient can influence the uptake, transport, and utilization of another. Knowledge of such interactions could promote the improvement of nutritional disorders, as well as the biofortification of elements such as Zn [34]. In addition, excessive accumulation of heavy metals such as Cu presents a critical parameter, as it may affect yield an quality, and it may also interact with nutrients such as N and Zn [26].

The present research examined different N levels in the NS of *S. cypria* grown in soilless cultivation, due to the importance of this essential element. In terms of plant growth and biomass production, previous findings indicate that *S. cypria* plants exhibit increased biomass production at moderate and elevated N levels (150–300 mg N L^−1^), compared to lower N levels (75 mg N L^−1^) [10]. In the present study, however, the application of various N levels did not affect the FW of *S. cypria* plants. This may be due to the seeds used to grow the experimental plants which originated from wild ecotypes, lacking breeding and selection for improved growth rates under high N availability. Stamnagathi (*Cichorium spinosum* L.) plants grown in an open hydroponic system, consisting of perlite as a growing media, exhibited a similar response, as increasing N levels from 4 to 16 mmol L^−1^ did not influence plant yield [35]. Nevertheless, the plants exhibited improved dry matter content under low and moderate N levels, in accordance with the findings of Chrysargyris and Tzortzakis [10]. Although fresh biomass production was unaffected, high N availability generally promotes increased cell expansion and subsequent leaf growth, as well as water uptake, negatively influencing DM [36]. This is a significant observation, as *S. cypria* is primarily purchased as dried material. Interestingly, excess Cu and Zn foliar applications did not have a significant effect on the plant growth of *S. cypria*. However, the combined application of increased Cu and foliar Zn led to a general decline in dry matter content. Similarly, in the study of Balamurugan et al. [37], a gradual increase in Cu applications resulted in a corresponding decrease in the dry matter content produced by green-gram (*Vigna radiata* (L.) Wilczek) plants. In addition, the application of excessive concentrations of Cu (60 μM), combined with beneficial or excessive concentrations of Zn (50 μM), significantly reduced the dry weight of *Arabidopsis thaliana* (L.) Heynh. plants. This was primarily attributed to the reduction in the activity of the photosynthetic apparatus, accompanied by a decrease in the relative total chlorophyll contents and chlorophyll fluorescence, caused by the excessive concentrations of Cu and Zn, applied individually or simultaneously [38]. Likewise, in the current study, low N levels (75 mg L^−1^) with excess Cu and foliar Zn caused a reduction in phytochemistry features such as SPAD, chlorophyll fluorescence, and chlorophyll content. However, under moderate N levels (150 mg L^−1^), the application of foliar Zn led to an increase in chlorophylls (a, b, and total). Likewise, Irmes et al. [39] observed a positive effect of foliar Zn application in the chlorophyll content of winter wheat (*Triticum aestivum* L.), contingent on the macronutrient levels utilized during fertilization, including N. This effect can be attributed to the essential role of Zn in chlorophyll biosynthesis and N metabolism, as well as its crucial function in facilitating N absorption, which indirectly contributes to enhanced chlorophyll content [29].

The different N levels applied in the current study, in conjunction with Cu and foliar Zn applications, impacted the nutrient content of *S. cypria* leaves and roots. The main finding indicates that, while the interaction between the three parameters did not significantly affect the nutrient content *S. cypria* leaves, it exerted a significant influence on root nutrient content and, specifically, the content of P, K, Na, Cu, and Zn. This is mainly attributed to the influence of N levels on uptake and mobility of macronutrients (P, K, and Na) and micronutrients (Cu and Zn) within the plants [40]. In addition, Cu may disrupt root cell membrane integrity, altering ionic homeostasis and disrupting ion selectivity mechanisms [41]. Supplementation of Zn also influences the uptake and translocation of macronutrients such as P and K, while it also competes with Cu for its utilization [42]. The N content in leaves and roots was positively influenced by the increased levels of N in the NS. Likewise, Chrysargyris and Tzortzakis [10] reported high N accumulation in *S. cypria* leaves, with the application of elevated N (300 mg L^−1^) in the NS. Similarly, in previous studies of MAPs, the N content of leaves and stems was enhanced by the increasing N supply in *Portulaca oleracea* L. and *Panax notoginseng* (Burkill) F. H. Chen [43,44]. Nitrogen contents in leaves and under moderate N fertilization (N150) were also marginally enhanced with the foliar application of Zn, as previously observed by Alinejad Elahshah et al. [45] in hydroponically grown strawberry (*Fragaria × ananassa* Duch.). The extent of the influence of Zn on shoot and root N uptake is directly related to the N availability during growth [46], with Zn improving the absorption capacity of N under recommended rates of N in maize (*Zea mays* L.) [47]. Owing to the mutual interaction between N and P nutrition in plants, an adequate supply of N has a positive effect in the uptake of P [48], while an excessive supply of N can reduce the uptake of P [49]. In the current study, increased N levels in the NS were linked to decreased accumulation of P in leaves and roots. This might be attributed to the plants’ ability to adjust their nutrient-allocation strategy in response to N addition [50]. Similarly, Li et al. [51] observed that N addition increased P limitation and demand for upper plant growth, affecting its concentrations in roots. In addition, K in roots was negatively influenced by the excessive Cu concentration in the NS, highlighting the influence of Cu toxicity in the uptake and accumulation of other nutrients. Similarly, the K content in roots of *Z. mays* L. was significantly reduced with the gradual increase in Cu to up to 78.7 μM in the NS [52]. Cu uptake generally depends on the plant species, organ, concentrations of Cu, and exposure duration. Saleem et al. [53] reported that flax (*Linum usitatissimum* L.) exposed to high Cu (up to 600 mg kg^−1^ soil) had initially accumulated Cu mainly in roots; meanwhile, following 140 DAS, Cu was mostly accumulated in leaf tissues. Similarly, in the current study, the application of 100 μM Cu in the NS increased its accumulation in *S. cypria* leaf tissues, compared to the application of 5 μM Cu, whereas its content in roots was relatively unaffected. This may be attributed to the duration of exposure, which allowed for the transportation to shoots, in line with the results obtained in earlier studies [53,54]. In addition, foliar Zn applications were able to reduce the accumulation of Cu in plant leaf tissues, at low and high N levels in the NS. This aligns with the findings of Shahriaripour and Tajabadipour [55], as the application of Zn adversely affected the uptake and leaf content of Cu in pistachio (*Pistacia vera* L.) seedlings. Nonetheless, the accumulation of Cu in leaves of *S. cypria* exposed to excess Cu in the NS aligns closely with previously reported findings under typical hydroponic cultivation [10], ranging from 17.3 to 22.2 mg Cu kg^−1^ DW under Cu excess. Moreover, these levels remain below the maximum residue levels of 100 mg kg^−1^ for Cu compounds in leaves and herbs for infusions, as outlined by EFSA [56]. Transition metals such as Cu, Zn, and Fe compete for their uptake, transport, and chemical reaction within the plant [57]. For instance, Cu above optimal concentrations may also influence Fe contents in plants due the rapid synthesis of Cu proteins that replace Fe proteins, reducing the demand for Fe [58]. In fact, in the current study, Fe was reduced in leaf tissues with the application of excess Cu; however, this was mostly evident with the combined application of high N (300 mg L^−1^) in the NS. For Zn, its foliar applications were anticipated to result in its accumulation in *S. cypria* plant tissues, as evidenced by Fatemi et al. [59] in pak choi (*Brassica rapa* L.) treated with foliar Zn at low (25 μΜ) and high (500 μM) concentrations. However, in the current study, a variable effect was observed, with Zn accumulation being contingent on N and Cu concentrations in the NS. This may be attributed to various factors such as Zn dosing, application frequency, and crop life cycle [60]. In addition, Zn optimization for its biofortification requires the optimization of N fertilization to ensure optimal synergy effects among the two nutrients [61]. Furthermore, proper N fertilization influences Zn cellular mobility and availability by its effects on Zn-chelating compounds such as amino acids and peptides. However, increased N supply may cause increased biomass production and decreased dry matter content, leading to a decrease in Zn concentration [47].

The biosynthesis of secondary metabolites, including phenolics, flavonoids, and antioxidants, is substantial in stress tolerance and protection from oxidative stress, inhibiting lipid peroxidation and neutralizing ROS [62,63]. It is generally accepted that the plant stress occurring from the excess of heavy metals prompts the antioxidant response of plants, with high levels of Cu prompting the increase in total phenolics in plants such as basil (*Ocimum basilicum* L.), common nettle (*Urtica dioica* L.), and *Acer platanoides* L. [25,62]. In addition, Zn can prompt the synthesis of phenolic compounds [25]. For instance, foliar applications of zinc oxide (ZnO) nanoparticles increased phenols, flavonoids, and antioxidant capacity according to ABTS, DPPH, and FRAP in habanero pepper plants (*Capsicum chinense* Jack.) [64]. Similarly, the foliar application of ZnSO_4_ increased total phenolics in *Lavandula stoechas* L. plants [65]. However, these responses, as observed by the researchers, were concentration-dependent. Interestingly, while discerning the main effects on the antioxidant status of *S. cypria* plants, Cu did not influence the content of phenolics and antioxidants (DPPH, FRAP, and ABTS). However, these indices were affected by the first- and second-order interactions of the examined parameters, suggesting that N, Cu, and Zn determine the antioxidant response of *S. cypria*. These coincides with the variation in total phenolics and antioxidant activity observed in the current study, which depended on the treatment applied. Particularly, foliar Zn applications increased total phenolics under moderate N levels (150 mg L^−1^), with similar response being observed for the antioxidant status of *S. cypria* plants, in terms of DPPH, FRAP, and ABTS. The response to Zn application is linked to the protective role of Zn, as an antioxidant cofactor, improving the synthesis of enzymatic and non-enzymatic antioxidants [66]. In addition, N levels may also exert influence on the antioxidant status of plants, especially under low or high concentrations [67]. However, differences were reported with the presence of increased Cu in the NS, possibly due to the influence of excess Cu on the synthesis and accumulation of antioxidant molecules.

A substantial effect of excess Cu is oxidative stress, attributed to the increased ROS production [63]. Rehman et al. [68] reported that the presence of excess Cu significantly increased leaf MDA contents of *Boehmeria nivea* L. plants, resulting in Cu-induced oxidative damage. However, the researchers also observed that this response was contingent on the level of N fertilization applied. Similarly, in the current study, MDA was relatively increased with the application of excess Cu concentrations when combined with 75 and 150 mg N L^−1^, corroborating with the notion that N fertilization modulates oxidative stress. In addition, Zn foliar application generally reduced the MDA content of *S. cypria*; however, this effect was subdued with the application of excess Cu in the NS. This can be attributed to the role of Zn in the maintenance of cell membrane integrity, and the activation of enzymes such as superoxide dismutase (SOD) and catalase (CAT) [66]. Similarly, in the current study, the application of foliar Zn decreased the content of H_2_O_2_, although this effect was limited to the application of the highest N concentration in the NS, as appropriate combined applications of N and Zn have a significant impact on the antioxidant enzyme activities to scavenge ROS as H_2_O_2_ [69].

## 4. Materials and Methods

### 4.1. Plant Materials and Experimental Site

The current study was conducted in a plastic multi-span greenhouse, located at the experimental farm of the Cyprus University of Technology, located in Kato Polemidia, Limassol, Cyprus, at a latitude of 34.700120 and a longitude of 32.984276. The experiment took place during the autumn–winter season. *S. cypria* seedlings were acquired from the Department of Aromatic Plants, Ministry of Agriculture, Cyprus. The seedlings were transplanted in 2.3 L pots in a 4:1 (*v*/*v*) mixture of coco soil and perlite, at the 7–10 leaf stage, on 25 November 2022. Each pot was accompanied by a plastic tray to collect runoff.

Plants were kept for 28 days after transplanting (DAT) in the same basic NS (sNS). Following this period, three different NS compositions were applied, consisting of the following N levels: (i) lower N, N75 (75 mg N L^−1^); (ii) intermediate (control) N, N150 (150 mg N L^−1^); and (iii) higher N, N300 (300 mg N L^−1^). These levels were determined based on previously reported data on the experimental evaluation of *S. cypria* [10,70]. Total N is the sum of NO_3_^−^-N and NH_4_^+^-N. All the other minerals were kept constant. The fertilizers used were calcium nitrate, potassium nitrate, ammonium nitrate, magnesium sulphate, magnesium nitrate, potassium sulphate, phosphoric acid, nitric acid, sulfuric acid, iron-chelate, manganese sulphate, zinc sulphate, copper sulphate, boric acid, and ammonium heptamolybdate. The electrical conductivity (EC) of the NS was maintained at 2.57 dS m^−1^, and the pH at 5.9 by adding HNO_3_. The composition of the different NS applied, including the sNS, is presented in Supplementary Table S1. Fertigation applications were determined based on real-time monitoring of weather conditions and substrate humidity. NSs were applied at a volume of 50–100 mL pot^−1^, with a frequency of one or two applications per week. Treatments were further diversified, considering the application of foliar spraying with pure dH_2_O (no foliar Zn) or dH_2_O with Zn (0.5 mg L^−1^ ZnSO_4_·7H_2_O or 1.74 mM Zn) (foliar Zn). Foliar was chosen, as it can more effectively be absorbed by and incorporated into plant tissues, resulting in rapidly improved micronutrient status, especially in the context of biofortification. In addition, short-term cultivation periods and successive harvesting of MAPs like *S. cypria* prompt swift and efficient supplementation methods. Foliar applications were conducted at three intervals, commencing at 58 DAT. The concentration, as well as the spraying interval, was based on preliminary findings [71]. At 58 DAT, plants were subjected to different Cu levels in the three NSs (N75, N150, and N300), namely (i) 5 μM Cu (control) and (ii) 100 μM Cu, with the addition of CuSO_4_. Plants were grown under these different Cu levels, with periodical fertigation applications every 2–3 days for an additional 42 days, resulting in a total growth duration of 100 DAT. During cultivation, common agricultural practices were followed. The average day and night temperatures were 19.5 °C and 13.6 °C, respectively, while the average day and night humidity values were 60.4% and 71.5%, respectively.

### 4.2. Agronomic Parameters

A total of 120 plants were used. Following 58 DAT of growth in the three different NS with 40 plants each, subsets of 20 plants were allocated to two different treatment groups: one group received the application of 5 μM Cu, while the other received 100 μM Cu in each NS. In addition, each subset was further partitioned to accommodate foliar applications as no foliar Zn applications, while the other was subjected to foliar Zn. Therefore, 12 treatments were used (3 N levels × 2 Cu levels × 2 Zn foliar applications), and each treatment accommodated 10 plants (replicates). Following cultivation, six plants for each treatment were considered for agronomic evaluation, and plant growth-related parameters were evaluated, including plant fresh (g) and dry weight (g), and dry matter content (%).

### 4.3. Leaf Phytochemistry Features

Prior to plant sampling, the relative chlorophyll content in terms of SPAD (Soil Plant Analysis Development) was assessed using an optical chlorophyll meter (SPAD-502, Minolta, Osaka, Japan). Leaf chlorophyll fluorescence (Fv Fm^−1^) was measured using OptiSci OS-30p Chlorophyll Fluorometer (Opti-Sciences, Hertfordshire, UK), while photosynthesis pigments (chlorophyll a, b, and total; and carotenoid content) were determined as previously described [72,73]. The ratios of Chla:Chlb and carotenoids/Total Chls were also calculated.

### 4.4. Plant-Tissue Ion Concentration Analysis

At the completion of the study, three leaf tissue samples were collected from a pool of plants per treatment and were dried at 42 °C. Dried leaf tissues were then ash-burned at 550 °C for four and a half hours and then acid-digested (2 M HCl). Potassium (K) and sodium (Na) were determined by flame photometry (Lasany Model 1832, Lasany International, Panchkula, India). Phosphorus (P) was determined using the molybdate/vanadate method (yellow method) by spectrophotometry (Multiskan GO, Thermo Fischer Scientific, Waltham, MA, USA). N was determined using the Kjeldahl method (BUCHI, Digest automat K-439 and Distillation Kjelflex K-360, Flawil, Switzerland), as described by Chrysargyris et al. [74]. Finally, iron (Fe), Zn, and Cu were determined by an atomic absorption spectrophotometer (PG Instruments AA500FG, Leicestershire, UK).

### 4.5. Total Phenols, Total Flavonoids, Antioxidants, and Stress Indicators

Methanolic extracts of leaf tissue (three replicates per treatment) were used for the determination of total phenols and flavonoids content. Total phenols content was determined by the Folin–Ciocâlteu assay at 755 nm. Results were expressed in gallic acid equivalents (mg GAE g^−1^ FW).

The antioxidant activity of the extracts was determined by the assays of 2,2-diphenyl-1-picrylhydrazyl (DPPH), ferric-reducing antioxidant power (FRAP), and the 2,2′-azino-bis(3-ethylbenzothiazoline-6-sulphonic acid) (ABTS) assay, as previously described [67]. DPPH radical scavenging activity was measured by bleaching with 0.3 M DPPH solution, where 1 mL of the solution was mixed with 15 μL leaf tissue extract and 1985 μL methanol (50%). Following incubation in the dark, the absorbance was measured at 517 nm (Multiskan GO, Thermo Fischer Scientific, Waltham, MA, USA). To measure FRAP, a FRAP solution was prepared with 2.5 mL CH_3_COONa·3H_2_O (0.3 mol L^−1^, pH 3.6), 0.25 mL TPTZ (Tripyridyl-s-triazine, 10 mmol L^−1^), FeCl_3_·10H_2_O (40 mmol L^−1^), and 15 μL leaf tissue extract. The absorbance was measured at 593 nm (Multiskan GO, Thermo Fischer Scientific, Waltham, MA, USA). Finally, for ABTS, 3 mL ABTS solution was added with 50 μL leaf tissue extract. Absorbance was measured at 734 nm. Results were expressed as Trolox (6-hydroxy-2,5,7,8-tetramethylchromane-2-carboxylic acid) equivalents (mg of Trolox g^−1^ FW).

Total flavonoids were determined by aluminum chloride colorimetric assay at 510 nm [67], and results were expressed in rutin equivalents (mg rutin g^−1^ FW).

Hydrogen peroxide (H_2_O_2_) and lipid peroxidation in terms of malondialdehyde (MDA) content were evaluated as described by Chrysargyris et al. [75]. Briefly, H_2_O_2_ was determined by measuring the absorbance at 390 nm, and results are expressed as μmol of H_2_O_2_ per gram of fresh weight (μmol H_2_O_2_ g^−1^ FW). MDA was determined by measuring the absorbance of reaction mixtures at 532 nm (corrected at 600 nm). MDA was calculated using the extinction coefficient of 155 mM^−1^ cm^−1^, and results are expressed as nmol of MDA per gram of fresh weight (nmol MDA g^−1^ FW).

### 4.6. Statistical Methods

Experimental data were analyzed by applying three-way analysis of variance (ANOVA) using SPSS v.21 (IBM, New York, NY, USA) to assess the main effects (N levels (N), Cu application (Cu), and Zn foliar application (Zn)), three first-order interactions (N × Cu, N × Zn, Cu × Zn), and a second-order interaction (N × Cu × Zn). Results are expressed as mean ± standard error (SE). Duncan’s multiple range tests were performed when ANOVA rendered a significant treatment impact at *p* < 0.05. The correlation coefficients between N levels in the NS, Cu levels in the NS, and foliar Zn application with individual parameters were tested by Pearson’s correlation test.

## 5. Conclusions

This study investigated the effects of different N levels (75, 150, and 300 mg L^−1^) in the NSs of *S. cypria* plants grown in soilless cultivation, in conjunction with normal (5 μM) and elevated (100 μM) Cu levels, with the aim of assessing the potential adverse effects of Cu toxicity. Moreover, foliar Zn was employed to examine their influence on plant nutritional status under varying NS regimes and their potential role in mitigating Cu-induced toxicity. Although the different N levels in the NSs did not affect the fresh biomass production of *S. cypria* plants, low (75 mg L^−1^) and moderate (150 mg L^−1^) N levels resulted in increased dry matter content. However, a general decline in dry mater content was observed with the combined application of foliar Zn and increased Cu, mainly due to the reduction in the activity of the photosynthetic apparatus. In addition, at moderate N (150 mg L^−1^), foliar Zn applications led to the increase in chlorophylls (a, b, and total). Increased concentrations of N in the NS facilitated the increase in the N content in leaves and roots, while foliar Zn enhanced the uptake of N under moderate N levels (150 mg L^−1^) in the NS. Excess Cu in the NS stimulated the accumulation of Cu in leaf tissues, while foliar Zn applications were able to reduce this accumulation at low (75 mg L^−1^) and high (150 mg L^−1^) N levels in the NS. At moderate N (150 mg L^−1^), foliar Zn applications enhanced total phenolics and antioxidants (DPPH, FRAP, and ABTS), with variations being observed depending on the N and Cu concentrations of the NS. In terms of stress response, MDA was increased with excess Cu at moderate-to-low N concentrations, while foliar Zn decreased both MDA and H_2_O_2_, contingent on Cu and N levels in the NS. The main outcome of this study is that exposure of *S. cypria* to excess Cu can impact the antioxidant activity and Cu content of *S. cypria* plants, with Zn alleviating potentially phytotoxic effects. N has a pivotal role in modulating these responses, while appropriate combined applications of N and Zn have a significant impact on the antioxidant response of plants and on the alleviation of Cu toxicity. Ultimately, low-to-moderate N levels in the NS can be applied under excess Cu conditions without impairing the growth, yield, and quality of *S. cypria* plants, and without compromising safety for consumption. In addition, Zn foliar applications can be applied as a means of modulating the response of plants under excess Cu, while also improving the quality of the final product.

## Figures and Tables

**Table 1:** The effect of N levels (N), Cu application (Cu), and Zn foliar application (Zn); their first-order interactions (N × Cu, N × Zn, and Cu × Zn); and their second-order interaction (N × Cu × Zn).

	N	Cu	Zn	N × Cu	N × Zn	Cu × Zn	N × Cu × Zn
FW (g)	ns	ns	ns	ns	ns	ns	ns
DM (%)	***	***	**	ns	ns	ns	ns
SPAD	ns	ns	ns	ns	ns	ns	ns
Fv Fm^−1^	***	***	ns	ns	**	ns	ns
Chl a (mg g^−1^)	*	ns	ns	**	***	*	***
Chl b (mg g^−1^)	ns	ns	ns	ns	**	ns	*
Total Chl (mg g^−1^)	*	ns	ns	*	***	*	**
Total Car (mg g^−1^)	*	ns	ns	***	***	*	***
Leaf N (g kg^−1^)	***	ns	**	***	**	ns	ns
Leaf P (g kg^−1^)	ns	ns	***	ns	***	ns	ns
Leaf K (g kg^−1^)	***	ns	ns	ns	ns	ns	ns
Leaf Na (g kg^−1^)	***	***	***	***	***	ns	ns
Leaf Fe (mg kg^−1^)	***	ns	ns	*	ns	ns	ns
Leaf Cu (mg kg^−1^)	ns	***	ns	ns	*	*	***
Leaf Zn (mg kg^−1^)	*	ns	ns	ns	*	ns	ns
Root N (g kg^−1^)	***	***	ns	***	***	***	ns
Root P (g kg^−1^)	***	ns	***	***	***	ns	*
Root K (g kg^−1^)	***	***	***	**	***	***	***
Root Na (g kg^−1^)	***	***	***	**	***	ns	***
Root Fe (mg kg^−1^)	ns	ns	*	ns	*	ns	ns
Root Cu (mg kg^−1^)	*	***	ns	**	*	ns	*
Root Zn (mg kg^−1^)	***	ns	**	**	***	ns	**
Phenols (mg GAE g^−1^)	***	ns	***	***	***	***	***
DPPH (mg Trolox g^−1^)	ns	ns	***	***	***	***	***
FRAP (mg Trolox g^−1^)	ns	ns	***	*	***	*	***
ABTS (mg Trolox g^−1^)	ns	ns	***	***	***	***	**
Flavonoids (mg Rutin g^−1^)	***	**	***	***	***	ns	***
H_2_O_2_ (μmol g^−1^)	***	ns	ns	*	***	ns	***
MDA (nmol g^−1^)	ns	Ns	***	***	ns	*	ns

*** Significant at *p* < 0.001; ** significant at *p* < 0.01; * significant at *p* < 0.05; ns, not significant, according to three-way ANOVA. Fresh weight (FW); dry matter (DM); chlorophyll fluorescence (Fv Fm^−1^) chlorophyll a (Chl a); Chlorophyll b (Chl b); total chlorophylls (Total Chl); total chlorophylls (Total Chl); total carotenoids (Total Car); 2,2-diphenyl-1-picrylhydrazyl (DPPH); ferric reducing antioxidant power (FRAP); 2,20-azino-bis(3-ethylbenzothiazoline-6-sulphonic acid) (ABTS); hydrogen peroxide (H_2_O_2_); malondialdehyde (MDA).

## Data Availability

The original contributions presented in this study are included in the article/supplementary material. Further inquiries can be directed to the corresponding author.

## Supplementary Materials

The following supporting information can be downloaded at https://www.mdpi.com/article/10.3390/plants14050691/s1, Table S1: Electrical conductivity (EC) and nutrient concentrations in the nutrient solution (NS) supplied to *S. cypria* plants grown in soilless cultivation under three levels in nitrogen; Table S2: Correlation coefficients and (*p*-values) between the N concentrations in the NS and the *Sideritis cypria* plant mineral content, growth, and physiology attributes; Table S3: Correlations coefficients and (*p*-values) between the Cu concentrations in the NS and the *Sideritis cypria* plant mineral content, growth, and physiology attributes; Table S4: Correlations coefficients and (*p*-values) between the Zn foliar application and the *Sideritis cypria* plant mineral content, growth, and physiology attributes.
