# Supplementary material for: Nitrogen Fertilization Coupled with Zinc Foliar Applications Modulate the Production, Quality, and Stress Response of Sideritis cypria Plants Grown Hydroponically Under Excess Copper Concentrations
Source: Plants (Basel). 2025 Feb 24;14(5):691. doi: 10.3390/plants14050691 (PMC11901989; doi:10.3390/plants14050691)
Supplement: Supplementary file 1 [file plants-14-00691-s001.zip › plants-3466821-supplementary.pdf]

Supplementary

Table S1. Electrical conductivity (EC) and nutrient concentrations in the nutrient solution (NS) supplied to *S. cypria* plants grown in soilless cultivation under three levels in nitrogen (N75: 75 mg L^{-1}, N150: 150 mg L^{-1}, N300: 300 mg L^{-1}).

**Parameters**	**S_{tarter} NS**	**N75**	**N150**	**N300**
EC dS m^{-1}	1.75	2.57	2.57	2.57
K^{+} mmol L^{-1}	7.55	8.95	8.95	8.95
Ca^{2+} mmol L^{-1}	3.50	3.74	3.74	3.74
Mg^{2+} mmol L^{-1}	1.00	2.88	2.88	2.88
NH_4^{+} mmol L^{-1}	0.5	0.62	0.62	0.62
NO_3^{-} mmol L^{-1}	13.72	5.35	10.71	21.42
SO_4^{2-} mmol L^{-1}	1.29	1.56	1.56	1.56
$H_2PO_4^{-}$ mmol L^{-1}	1.80	2.42	2.42	2.42
Cl^{-} mmol L^{-1}	1.49	1.49	1.49	1.49
Fe μmol L^{-1}	30.00	71.56	71.56	71.56
Mn μmol L^{-1}	5.00	18.21	18.21	18.21
Zn μmol L^{-1}	4.00	1.53	1.53	1.53
Cu μmol L^{-1}	1.00	4.72	4.72	4.72
B μmol L^{-1}	30.00	18.52	18.52	18.52
Mo μmol L^{-1}	0.50	0.52	0.52	0.52
N/K		0.64	1.27	2.53
K/Total N		1.57	0.79	0.39
K/(K+Ca+Mg)		0.40	0.40	0.40
Ca/(K+Ca+Mg)		0.34	0.34	0.34
Mg/(K+Ca+Mg)		0.26	0.26	0.26

Table S2. Correlations coefficients and (p-values) between the N concentrations in the NS and the *Sideritis cypria* plant mineral content, growth, and physiology attributes.

		N levels	LeafN	LeafP	LeafNa	LeafK	LeafFe	LeafCu	LeafZn	RootN	RootP	RootNa	RootK	RootFe	RootCu	RootZn
**N levels**	r	1	0.688**	-0.026	0.653**	0.023	-0.526**	0.047	0.333*	0.499**	-0.367*	0.292	-0.552**	0.290	0.267	0.067
	p		0.000	0.881	0.000	0.892	0.001	0.783	0.021	0.002	0.028	0.084	0.000	0.086	0.116	0.700
**SPAD**	r	-0.150	0.005	-0.124	-0.070	0.033	0.036	0.007	0.035	0.040	-0.053	-0.043	-0.074	0.039	0.029	-0.063
	p	0.209	0.952	0.168	0.439	0.711	0.685	0.936	0.664	0.660	0.554	0.634	0.411	0.666	0.747	0.485
**Fv/Fm**	r	-0.046	-0.165	-0.127	0.015	0.113	0.412**	0.160	-0.165	0.229**	0.574**	0.023	0.026	-0.321**	-0.058	0.321**
	p	0.789	0.064	0.159	0.871	0.210	0.000	0.073	0.076	0.010	0.000	0.802	0.775	0.000	0.515	0.000
**FW**	r	0.160	0.063	0.161	0.227*	0.054	-0.243**	-0.214*	-0.031	0.198*	-0.195*	0.303**	-0.171	-0.016	-0.140	0.085
	p	0.271	0.483	0.073	0.011	0.551	0.006	0.016	0.708	0.026	0.028	0.001	0.055	0.858	0.118	0.345
**DM**	r	-0.612**	-0.301**	0.053	-0.515**	-0.123	0.065	-0.069	0.200*	-0.405**	0.307**	-0.110	0.409**	0.307**	0.009	-0.219*
	p	0.000	0.001	0.561	0.000	0.173	0.472	0.446	0.031	0.000	0.000	0.220	0.000	0.000	0.919	0.014
**Chl a**	r	0.000	0.098	0.031	-0.224*	-0.176*	-0.152	0.089	0.171	-0.157	-0.008	-0.045	0.212*	0.045	0.185*	-0.149
	p	1.000	0.277	0.728	0.012	0.050	0.090	0.324	0.067	0.079	0.931	0.618	0.017	0.620	0.038	0.096
**Chl b**	r	-0.070	0.140	0.037	-0.191*	-0.185*	-0.094	0.101	0.138	-0.106	-0.058	0.081	0.200*	0.112	0.214*	-0.173
	p	0.685	0.117	0.681	0.033	0.039	0.295	0.261	0.140	0.239	0.516	0.370	0.025	0.213	0.016	0.052
**Tot chl**	r	-0.022	0.119	0.032	-0.215*	-0.188*	-0.139	0.097	0.164	-0.143	-0.031	0.000	0.213*	0.072	0.204*	-0.163
	p	0.900	0.185	0.724	0.016	0.036	0.120	0.280	0.078	0.111	0.732	0.999	0.017	0.426	0.022	0.068
**Tot car**	r	0.185	0.050	0.116	-0.108	0.039	-0.126	0.063	0.134	-0.052	-0.016	-0.066	0.119	-0.059	0.177*	-0.095
	p	0.281	0.580	0.198	0.232	0.666	0.161	0.483	0.152	0.562	0.859	0.460	0.183	0.510	0.048	0.289
**Phenols**	r	-0.070	-0.272**	-0.051	-0.285**	0.110	0.232**	0.038	-0.074	-0.106	0.198*	-0.483**	0.021	-0.198*	-0.084	0.021
	p	0.685	0.002	0.570	0.001	0.222	0.009	0.672	0.427	0.236	0.026	0.000	0.818	0.026	0.352	0.814
**DPPH**	r	-0.013	-0.153	-0.178*	-0.265**	0.096	0.232**	0.072	0.051	-0.082	0.174	-0.398**	0.029	-0.056	-0.115	0.042
	p	0.939	0.086	0.046	0.003	0.287	0.009	0.420	0.586	0.363	0.052	0.000	0.748	0.533	0.201	0.641
**FRAP**	r	-0.134	-0.141	-0.043	-0.127	0.188*	0.201*	0.091	-0.146	0.131	0.154	-0.294**	-0.049	-0.360**	0.011	0.059
	p	0.438	0.116	0.632	0.158	0.035	0.024	0.309	0.118	0.145	0.086	0.001	0.587	0.000	0.906	0.511
**ABTS**	r	-0.047	-0.163	-0.077	-0.075	0.058	0.180*	-0.108	0.008	0.031	0.059	-0.265**	-0.120	-0.097	-0.096	0.072
	p	0.785	0.068	0.390	0.405	0.521	0.043	0.228	0.932	0.731	0.508	0.003	0.179	0.282	0.285	0.422
**Flavonoids**	r	-0.002	0.089	-0.180*	-0.215*	-0.029	0.110	0.050	0.147	-0.101	-0.120	-0.101	-0.107	0.066	-0.068	0.009
	p	0.993	0.320	0.045	0.016	0.749	0.221	0.576	0.115	0.261	0.182	0.259	0.234	0.464	0.448	0.917
**H2O2**	r	-0.387*	-0.231**	-0.057	-0.228*	0.068	0.111	0.076	-0.072	-0.138	0.198*	-0.198*	0.401**	-0.136	-0.022	0.005
	p	0.020	0.009	0.529	0.011	0.451	0.216	0.396	0.443	0.124	0.026	0.026	0.000	0.128	0.808	0.955
**MDA**	r	-0.066	0.106	0.241**	-0.178*	-0.073	-0.084	0.077	-0.104	-0.357**	0.045	-0.071	0.227*	0.245**	0.107	-0.289**
	p	0.703	0.237	0.007	0.047	0.420	0.351	0.391	0.266	0.000	0.620	0.427	0.010	0.006	0.235	0.001

**. Correlation is significant at the 0.01 level (2-tailed).; *. Correlation is significant at the 0.05 level (2-tailed).

Table S3. Correlations coefficients and (p-values) between the Cu concentrations in the NS and the *Sideritis cypria* plant mineral content, growth, and physiology attributes.

		Cu	LeafN	LeafP	LeafNa	LeafK	LeafFe	LeafCu	LeafZn	RootN	RootP	RootNa	RootK	RootFe	RootCu	RootZn
**Cu**	r	1	0.051	-0.090	0.289	0.094	-0.087	0.871**	-0.171	-0.123	-0.052	-0.293	-0.301	-0.087	0.514**	0.108
	p		0.767	0.600	0.087	0.585	0.612	0.000	0.245	0.474	0.763	0.083	0.074	0.613	0.001	0.532
**SPAD**	r	0.038	0.005	-0.124	-0.070	0.033	0.036	0.007	0.035	0.040	-0.053	-0.043	-0.074	0.039	0.029	-0.063
	p	0.749	0.952	0.168	0.439	0.711	0.685	0.936	0.664	0.660	0.554	0.634	0.411	0.666	0.747	0.485
**Fv/Fm**	r	-0.409*	-0.165	-0.127	0.015	0.113	0.412**	0.160	-0.165	0.229**	0.574**	0.023	0.026	-0.321**	-0.058	0.321**
	p	0.013	0.064	0.159	0.871	0.210	0.000	0.073	0.076	0.010	0.000	0.802	0.775	0.000	0.515	0.000
**FW**	r	-0.048	0.063	0.161	0.227*	0.054	-0.243**	-0.214*	-0.031	0.198*	-0.195*	0.303**	-0.171	-0.016	-0.140	0.085
	p	0.743	0.483	0.073	0.011	0.551	0.006	0.016	0.708	0.026	0.028	0.001	0.055	0.858	0.118	0.345
**DM**	r	-0.340*	-0.301**	0.053	-0.515**	-0.123	0.065	-0.069	0.200*	-0.405**	0.307**	-0.110	0.409**	0.307**	0.009	-0.219*
	p	0.042	0.001	0.561	0.000	0.173	0.472	0.446	0.031	0.000	0.000	0.220	0.000	0.000	0.919	0.014
**Chl a**	r	0.032	0.098	0.031	-0.224*	-0.176*	-0.152	0.089	0.171	-0.157	-0.008	-0.045	0.212*	0.045	0.185*	-0.149
	p	0.853	0.277	0.728	0.012	0.050	0.090	0.324	0.067	0.079	0.931	0.618	0.017	0.620	0.038	0.096
**Chl b**	r	0.065	0.140	0.037	-0.191*	-0.185*	-0.094	0.101	0.138	-0.106	-0.058	0.081	0.200*	0.112	0.214*	-0.173
	p	0.705	0.117	0.681	0.033	0.039	0.295	0.261	0.140	0.239	0.516	0.370	0.025	0.213	0.016	0.052
**Tot chl**	r	0.052	0.119	0.032	-0.215*	-0.188*	-0.139	0.097	0.164	-0.143	-0.031	0.000	0.213*	0.072	0.204*	-0.163
	p	0.764	0.185	0.724	0.016	0.036	0.120	0.280	0.078	0.111	0.732	0.999	0.017	0.426	0.022	0.068
**Tot car**	r	0.050	0.050	0.116	-0.108	0.039	-0.126	0.063	0.134	-0.052	-0.016	-0.066	0.119	-0.059	0.177*	-0.095
	p	0.771	0.580	0.198	0.232	0.666	0.161	0.483	0.152	0.562	0.859	0.460	0.183	0.510	0.048	0.289
**Phenols**	r	0.103	-0.272**	-0.051	-0.285**	0.110	0.232**	0.038	-0.074	-0.106	0.198*	-0.483**	0.021	-0.198*	-0.084	0.021
	p	0.551	0.002	0.570	0.001	0.222	0.009	0.672	0.427	0.236	0.026	0.000	0.818	0.026	0.352	0.814
**DPPH**	r	0.017	-0.153	-0.178*	-0.265**	0.096	0.232**	0.072	0.051	-0.082	0.174	-0.398**	0.029	-0.056	-0.115	0.042
	p	0.921	0.086	0.046	0.003	0.287	0.009	0.420	0.586	0.363	0.052	0.000	0.748	0.533	0.201	0.641
**FRAP**	r	0.028	-0.141	-0.043	-0.127	0.188*	0.201*	0.091	-0.146	0.131	0.154	-0.294**	-0.049	-0.360**	0.011	0.059
	p	0.872	0.116	0.632	0.158	0.035	0.024	0.309	0.118	0.145	0.086	0.001	0.587	0.000	0.906	0.511
**ABTS**	r	-0.057	-0.163	-0.077	-0.075	0.058	0.180*	-0.108	0.008	0.031	0.059	-0.265**	-0.120	-0.097	-0.096	0.072
	p	0.743	0.068	0.390	0.405	0.521	0.043	0.228	0.932	0.731	0.508	0.003	0.179	0.282	0.285	0.422
**Flavonoids**	r	-0.145	0.089	-0.180*	-0.215*	-0.029	0.110	0.050	0.147	-0.101	-0.120	-0.101	-0.107	0.066	-0.068	0.009
	p	0.397	0.320	0.045	0.016	0.749	0.221	0.576	0.115	0.261	0.182	0.259	0.234	0.464	0.448	0.917
**H2O2**	r	-0.107	-0.231**	-0.057	-0.228*	0.068	0.111	0.076	-0.072	-0.138	0.198*	-0.198*	0.401**	-0.136	-0.022	0.005
	p	0.534	0.009	0.529	0.011	0.451	0.216	0.396	0.443	0.124	0.026	0.026	0.000	0.128	0.808	0.955
**MDA**	r	0.080	0.106	0.241**	-0.178*	-0.073	-0.084	0.077	-0.104	-0.357**	0.045	-0.071	0.227*	0.245**	0.107	-0.289**
	p	0.641	0.237	0.007	0.047	0.420	0.351	0.391	0.266	0.000	0.620	0.427	0.010	0.006	0.235	0.001

**. Correlation is significant at the 0.01 level (2-tailed).; *. Correlation is significant at the 0.05 level (2-tailed).

Table S4. Correlations coefficients and (p-values) between the Zn foliar application and the *Sideritis cypria* plant mineral content, growth, and physiology attributes.

		**Foliar**	**LeafN**	**LeafP**	**LeafNa**	**LeafK**	**LeafFe**	**LeafCu**	**LeafZn**	**RootN**	**RootP**	**RootNa**	**RootK**	**RootFe**	**RootCu**	**RootZn**
**Foliar**	**r**	1	0.119	0.500**	-0.190	0.043	-0.027	-0.014	-0.171	0.037	-0.138	-0.304	0.156	-0.319	-0.087	-0.266
	**p**		0.491	0.002	0.268	0.803	0.875	0.936	0.245	0.829	0.424	0.072	0.362	0.058	0.615	0.116
**SPAD**	**r**	0.012	0.005	-0.124	-0.070	0.033	0.036	0.007	0.035	0.040	-0.053	-0.043	-0.074	0.039	0.029	-0.063
	**p**	0.921	0.952	0.168	0.439	0.711	0.685	0.936	0.664	0.660	0.554	0.634	0.411	0.666	0.747	0.485
**Fv/Fm**	**r**	-0.180	-0.165	-0.127	0.015	0.113	0.412**	0.160	-0.165	0.229**	0.574**	0.023	0.026	-0.321**	-0.058	0.321**
	**p**	0.294	0.064	0.159	0.871	0.210	0.000	0.073	0.076	0.010	0.000	0.802	0.775	0.000	0.515	0.000
**FW**	**r**	-0.064	0.063	0.161	0.227*	0.054	-0.243**	-0.214*	-0.031	0.198*	-0.195*	0.303**	-0.171	-0.016	-0.140	0.085
	**p**	0.660	0.483	0.073	0.011	0.551	0.006	0.016	0.708	0.026	0.028	0.001	0.055	0.858	0.118	0.345
**DM**	**r**	-0.319	-0.301**	0.053	-0.515**	-0.123	0.065	-0.069	0.200*	-0.405**	0.307**	-0.110	0.409**	0.307**	0.009	-0.219*
	**p**	0.058	0.001	0.561	0.000	0.173	0.472	0.446	0.031	0.000	0.000	0.220	0.000	0.000	0.919	0.014
**Chl a**	**r**	0.155	0.098	0.031	-0.224*	-0.176*	-0.152	0.089	0.171	-0.157	-0.008	-0.045	0.212*	0.045	0.185*	-0.149
	**p**	0.365	0.277	0.728	0.012	0.050	0.090	0.324	0.067	0.079	0.931	0.618	0.017	0.620	0.038	0.096
**Chl b**	**r**	0.188	0.140	0.037	-0.191*	-0.185*	-0.094	0.101	0.138	-0.106	-0.058	0.081	0.200*	0.112	0.214*	-0.173
	**p**	0.272	0.117	0.681	0.033	0.039	0.295	0.261	0.140	0.239	0.516	0.370	0.025	0.213	0.016	0.052
**Tot chl**	**r**	0.168	0.119	0.032	-0.215*	-0.188*	-0.139	0.097	0.164	-0.143	-0.031	0.000	0.213*	0.072	0.204*	-0.163
	**p**	0.328	0.185	0.724	0.016	0.036	0.120	0.280	0.078	0.111	0.732	0.999	0.017	0.426	0.022	0.068
**Tot car**	**r**	0.101	0.050	0.116	-0.108	0.039	-0.126	0.063	0.134	-0.052	-0.016	-0.066	0.119	-0.059	0.177*	-0.095
	**p**	0.560	0.580	0.198	0.232	0.666	0.161	0.483	0.152	0.562	0.859	0.460	0.183	0.510	0.048	0.289
**Phenols**	**r**	0.445**	-0.272**	-0.051	-0.285**	0.110	0.232**	0.038	-0.074	-0.106	0.198*	-0.483**	0.021	-0.198*	-0.084	0.021
	**p**	0.007	0.002	0.570	0.001	0.222	0.009	0.672	0.427	0.236	0.026	0.000	0.818	0.026	0.352	0.814
**DPPH**	**r**	0.323	-0.153	-0.178*	-0.265**	0.096	0.232**	0.072	0.051	-0.082	0.174	-0.398**	0.029	-0.056	-0.115	0.042
	**p**	0.054	0.086	0.046	0.003	0.287	0.009	0.420	0.586	0.363	0.052	0.000	0.748	0.533	0.201	0.641
**FRAP**	**r**	0.415*	-0.141	-0.043	-0.127	0.188*	0.201*	0.091	-0.146	0.131	0.154	-0.294**	-0.049	-0.360**	0.011	0.059
	**p**	0.012	0.116	0.632	0.158	0.035	0.024	0.309	0.118	0.145	0.086	0.001	0.587	0.000	0.906	0.511
**ABTS**	**r**	0.470**	-0.163	-0.077	-0.075	0.058	0.180*	-0.108	0.008	0.031	0.059	-0.265**	-0.120	-0.097	-0.096	0.072
	**p**	0.004	0.068	0.390	0.405	0.521	0.043	0.228	0.932	0.731	0.508	0.003	0.179	0.282	0.285	0.422
**Flavonoids**	**r**	0.309	0.089	-0.180*	-0.215*	-0.029	0.110	0.050	0.147	-0.101	-0.120	-0.101	-0.107	0.066	-0.068	0.009
	**p**	0.066	0.320	0.045	0.016	0.749	0.221	0.576	0.115	0.261	0.182	0.259	0.234	0.464	0.448	0.917
**H2O2**	**r**	0.068	-0.231**	-0.057	-0.228*	0.068	0.111	0.076	-0.072	-0.138	0.198*	-0.198*	0.401**	-0.136	-0.022	0.005
	**p**	0.692	0.009	0.529	0.011	0.451	0.216	0.396	0.443	0.124	0.026	0.026	0.000	0.128	0.808	0.955
**MDA**	**r**	-0.556**	0.106	0.241**	-0.178*	-0.073	-0.084	0.077	-0.104	-0.357**	0.045	-0.071	0.227*	0.245**	0.107	-0.289**
	**p**	0.000	0.237	0.007	0.047	0.420	0.351	0.391	0.266	0.000	0.620	0.427	0.010	0.006	0.235	0.001

**. Correlation is significant at the 0.01 level (2-tailed).; *. Correlation is significant at the 0.05 level (2-tailed).
